# Overexpression of the ATP binding cassette gene ABCA1 determines resistance to Curcumin in M14 melanoma cells

**DOI:** 10.1186/1476-4598-8-129

**Published:** 2009-12-23

**Authors:** Beatrice E Bachmeier, Cristina M Iancu, Peter H Killian, Emanuel Kronski, Valentina Mirisola, Giovanna Angelini, Marianne Jochum, Andreas G Nerlich, Ulrich Pfeffer

**Affiliations:** 1 Department of Clinical Chemistry and Clinical Biochemistry, Surgical Hospital, Ludwig-Maximilians-University Munich, Munich, Germany; 2 Department of Biochemistry, University Bucharest, Romania; 3 Institute of Pathology, Academic Hospital Munich-Bogenhausen, Munich, Germany; 4 Functional Genomics, National Cancer Research Institute, Genova, Italy

## Abstract

**Background:**

Curcumin induces apoptosis in many cancer cells and it reduces xenograft growth and the formation of lung metastases in nude mice. Moreover, the plant derived polyphenol has been reported to be able to overcome drug resistance to classical chemotherapy. These features render the drug a promising candidate for tumor therapy especially for cancers known for their high rates concerning therapy resistance like melanoma.

**Results:**

We show here that the melanoma cell line M14 is resistant to Curcumin induced apoptosis, which correlates with the absence of any effect on NFκB signaling. We show that CXCL1 a chemokine that is down regulated in breast cancer cells by Curcumin in an NFκB dependant manner is expressed at variable levels in human melanomas. Yet in M14 cells, CXCL1 expression did not change upon Curcumin treatment. Following the hypothesis that Curcumin is rapidly removed from the resistant cells, we analyzed expression of known multi drug resistance genes and cellular transporters in M14 melanoma cells and in the Curcumin sensitive breast cancer cell line MDA-MB-231. ATP-binding cassette transporter ABCA1, a gene involved in the cellular lipid removal pathway is over-expressed in resistant M14 melanoma as compared to the sensitive MDA-MB-231 breast cancer cells. Gene silencing of ABCA1 by siRNA sensitizes M14 cells to the apoptotic effect of Curcumin most likely as a result of reduced basal levels of active NFκB. Moreover, ABCA1 silencing alone also induces apoptosis and reduces p65 expression.

**Conclusion:**

Resistance to Curcumin thus follows classical pathways and ABCA1 expression should be considered as response marker.

## Bachground

The naturally occurring polyphenol Curcumin (Diferuloylmethane) is a major component of the rhizome of tumeric (*Curcuma longa*) and commonly used as a spice in India. It has been used for centuries as a traditional medicine to treat various inflammatory disorders [1,2] and has revealed remarkable anti-tumor activity in various organs and cell models [3-11]). offering a role as novel candidate for chemoprevention of cancer.

In a previous study we have shown that Curcumin significantly reduces the number of metastases formed from intracardially injected breast cancer cells [3]. The underlying molecular mechanism involves the inhibition of the survival related transcription factor nuclear factor κB (NFκB) and its down-stream targets, the pro-inflammatory cytokines CXCL1 and -2 [4]. CXCL1 and -2 are expressed at high levels in very few breast cancers but in many primary and metastatic melanomas (see results section). We therefore addressed the question whether Curcumin could be a possible candidate drug for the chemoprevention of this type of cancer.

Melanoma, the most deadly form of skin cancer, is very aggressive and resistant to present therapies. The current treatment modalities for melanoma fail to prevent the spread of metastasis in nearly 50% of the patients [12]. The development of new therapies is therefore required.

The use of Curcumin in *in vitro *models of melanoma has so far shown promising results, inasmuch as the melanoma cell lines responded well to the polyphenol in terms of diminished NFκB activity, which is associated with reduced proliferation and induction of apotosis [13,14].

However, the cell lines used in these studies are less tumorigenic and have a lower metastatic potential than the highly metastatic human M14 melanoma cell line that we use here [15-17].

Curcumin acts at least in part through diminished translocation of the transcription factor NFκB [18] which is constitutively active in many tumor cells. Inhibition of NFκB activity is associated with anti-proliferative effects, as well as with the induction of apoptosis [19,20]. Several signal transduction pathways converge on NFκB and its regulators to mediate the transcriptional control of apoptosis and cell-cycle control [21,22]. NFκB is required for prevention of cell death induced by tumor necrosis factor alpha (TNF-alpha) and its ability to induce anti-apoptotic genes such as *bcl2 *and *birc5/survivin *protects cancer cells from apoptosis [23,24]. Activation of NFκB constitutes a crucial step in tumor promotion and progression, angiogenesis, inflammation, invasion, and metastasis [25].

In the present investigation, we show that M14 cells are resistant to the apoptotic effects of Curcumin and demonstrate that constitutively active pro-apoptotic NFκB cannot be inactivated by the polyphenol in these highly metastatic melanoma cells. As a consequence, the polyphenol does not reduce the expression of the metastasis-related pro-inflammatory cytokine CXCL1/GROα [26], which is known to be a NFκB target [27].

Drug resistance is most often determined by over-expression of multidrug resistance genes, a superfamily of transmembrane proteins that act as ATP hydrolyzing cellular transporters and are able to export a wide variety of natural and synthetic compounds from the cells (for a review see [28]).

Here we show that resistance of M14 melanoma cells to Curcumin is due to over-expression of the cholesterol transporter ABCA1 and demonstrate that silencing of this multi-drug resistance gene leads to better response of M14 cells to the polyphenol.

## Materials and methods

### Cell Types and Culture Conditions

M14 human melanoma cell line was cultured in RPMI-1640 medium supplemented with fetal calf serum (10%), gentamycin (0,1%) and L-glutamine (1%). MDA-MB-231 human breast cancer cells were grown in MEM (Eagle's) with Earle's salts supplemented with 5% heat inactivated fetal calf serum, 1% L-glutamine solution (200 mM), 1% sodium pyruvate solution (100 mM), non-essential amino acids and vitamins. Cells were grown at 37°C in a humidified atmosphere of 5% CO_2_. Medium was changed every two days.

### Curcumin Treatment of Cells

Curcumin with a purity of 95% was purchased from Fluka (Buchs, Switzerland), dissolved in 0.5 M NaOH as a 25 mM stock solution and stored at -20°C. For the use in cell culture a 2.5 mM solution in sterile PBS was prepared. Curcumin was applied at an end concentration of 25 μM for all assays.

### Flow Cytometric Analysis

Human M14 melanoma cells were cultivated in a 24 well-plate and incubated with 25 μM Curcumin for various time intervals. The cells were harvested and then treated with (FITC)-conjugated annexin V and propidium iodide (Annexin-V-FLUOS Staining kit from Roche, Germany) according to the recommendations of the manufacturer. Ten thousand events were counted for each sample. Data were analyzed using a Flow-Cytometer (Beckman Coulter XL-MCL, Software: System II).

### Electrophoretic Mobility Shift Assay

M14 cells were seeded onto 150 cm^2 ^culture dishes with 25 ml culture medium and treated for various time intervals (2, 4, 6 h) with 25 μM Curcumin. Nuclear extracts were prepared as described previously [29]. Oligonucleotides corresponding to the consensus sequences of NFκB (5'-GTTAGTTGA**GGGGACTTT**CCCA-GGC-3') were labeled with [alpha-^32^P]dATP (3000 Ci/mM) and Klenow enzyme and were incubated with 10 μg of nuclear protein in 20 μl of 7 mM Hepes-KOH (pH 7.9), 100 mM KCl, 3.6 mM MgCl_2_, and 10% glycerol on ice for 20 min. Poly [d(I-C)] (0.05 mg/ml) was added as an unspecific competitor. The samples were run on a 5% nondenaturing polyacrylamide gel in a buffer containing 25 mM Tris-HCl (pH 8.0), 190 mM glycine, and 1 mM EDTA. Gels were dried and analyzed by autoradiography. In order to prove the specificity of the probes, a 50 fold excess of unlabeled probe was incubated with the binding reaction mixture for 45 min on ice before adding the radiolabeled DNA fragment.

### Immunocytochemistry for NFκB p65 Location

The nuclear translocation of p65 was examined by immunocytochemistry as described previously [30,31]. Briefly, cells were plated on SuperFrost glass slides for adherence and treated the next day with Curcumin. Slides were air-dried for 1 h at room temperature and fixed with ice-cold acetone. After brief washing in PBS, slides were blocked with a blocking solution (Biogenex, San Ramon, CA) for 1 h and then incubated with a 1:100 dilution of rabbit polyclonal anti-human p65 antibody (Santa Cruz Biotechnology, Santa Cruz, CA). After overnight incubation, the slides were washed and then incubated with goat anti-rabbit IgG-Alexa 594 (Invitrogen - Molecular Probes, Carlsbad, CA) for 1 h and counter-stained for nuclei with 1 μg/ml 4',6-diamidino-2-phenylindole (DAPI) for 5 min. Stained slides were mounted with mounting medium (Vector Labs, Burlingame, CA) and analyzed under a fluorescence microscope with digital image capture (Leica, Bensheim, Germany).

### Gene Silencing

RNA interference (RNAi) was used to generate specific knockdowns of ABCA1 mRNA transcripts in the human melanoma cell line M14. Small interfering RNAs targeted to ABCA1 were purchased pre-designed (Ambion, UK). A non-silencing fluorescein labeled siRNA (Qiagen, Hilden, Germany) was used as control for transfection efficiency as well as for monitoring the effect of silencing during all experiments. Cell cultures with at least 90% transfection efficiency were used for further studies. Transfection of M14 cells (40% confluency) with siRNA was performed using Lipofectamine 2000 (Invitrogen, Carlsbad, CA) according to the recommendations of the manufacturer. Briefly, the transfection reagent was pre-incubated with the siRNA Oligos either targeted to ABCA1 or to an irrelevant control 30 min prior to the application to the cells. Cells were harvested at various time points between 2 h and 24 h after transfection.

### Quantitative RT-PCR

Expression data validation was performed by quantitative real-time RT-PCR using the RNA extracted from drug- or mock-treated cells and analysis of transcript expression after gene silencing was performed from cells transfected with siRNAs or a non-silencing fluorescein labeled control. RNA was extracted from cells using the RNeasy Protect Mini Kit (Qiagen, Hilden, Germany) according to the recommendation of the manufacturer and reverse transcribed as above with oligo dT primers in 20 μl final volume. All primers for the genes tested were designed using primer3 software [32] with a Tm optimum of approximately 60°C and a product length of 100-150 nt (see additional file 1). Real time PCR was performed on an I-Cycler (Biorad Hercules, CA) using iQ Supermix (Biorad) supplemented with 10 nM fluorescein (Biorad), 0.1× Sybr-Green I (Sigma-Aldrich), 2.5 μl of cDNA (5× diluted), 3 pmol sense and antisense primers in a final reaction volume of 25 μl. After an initial denaturation step of 3 min, 50 cycles of 15 sec at 95°C followed by 30 sec at 60°C were performed. Fluorescence was measured during the annealing step in each cycle. After amplification melting curves with 80 steps of 15 sec and 0.5°C increase were performed to monitor amplicon identity. Amplification efficiency was assessed for all primer sets utilized in separate reactions, and primers with efficiencies >94% were used. Expression data were normalized on HPRT and on RNA polymerase II (RPII) gene expression data obtained in parallel. Relative expression values with standard errors were obtained using Qgene software [33] and statistical comparisons (unpaired two-tailed t-test) were performed using Prism (GraphPad) software. Expression changes were calculated using the mean value of normalizations obtained using HPRT and RPII genes as references.

### Protein Determination

Protein concentrations were determined by the BCA protein assay (Pierce, Oud-Beijerland, Netherlands) with bovine serum albumin as the standard.

### Western Blots

Conditioned media from Curcumin-treated (6, 15 and 24 h) and non-treated control cells were analyzed using an antibody against CXCL1 (Dianova, Hamburg, Germany). Equal amounts of protein were subjected to sodium dodecyl sulfate-polyacrylamide gel electrophoresis and the amount of protein blotted onto the membranes was visualized with Ponceau red before blocking. Following electrophoretic separation by sodium dodecyl sulfate-polyacrylamide gel electrophoresis, proteins were electroblotted on nitrocellulose membranes (Whatman, Brentford, UK). The membranes were blocked in 5% non-fat milk (Merck, Darmstadt, Germany) overnight at 4°C. The first antibody was incubated for 1 h at room temperature. Thereafter, membranes were washed in tris buffered saline with Tween buffer, and a further incubation was carried out with a peroxidase-conjugated antibody (Dako, Hamburg, Germany) for 1 h at room temperature. The enhanced chemiluminescence system was used for visualization of the protein bands as recommended by the manufacturer (GE Healthcare, Little Chalfont, UK).

### Apoptosis Assay

Apoptotic cell death was determined by an enzyme-linked immunoassay (Cell Death Detection ELISA ^PLUS^, Roche) to detect fragmented DNA and histones (mononucleosomes and oligonucleosomes). Human melanoma cells M14 were seeded on 96-well plates and silenced by transfection with siRNAs directed against ABCA1 or a nonsense targeted sequence in combination with 25 μM Curcumin or without treatment. Lysates prepared from the cells were processed following the instructions of the manufacturer

### Microarray gene expression analyses

Total RNA was extracted from M14 melanoma and MDA-MB-231 breast cancer cells as described above. Sample preparation, hybridization and scanning of Affymetrix (Santa Clara, CA, USA) HGU133plus2 arrays were performed following the instructions given by the provider. Raw expression data were pre-processed using the GCRMA algorithm implemented in R/Bioconductor. Genes with expression values below an unlogged intensity of 100 in both cell lines were not considered. Significance Analysis of Microarrays [34] was used for statistical evaluation of differential gene expression values.

Micorarray gene expression dataset GSE7553 [35] were obtained form Gene Expression Omnibus http://www.ncbi.nlm.nih.gov/geo/. The expression values published by the authors were used without further processing. Statistical considerations were done using Prism Graphpad software.

## Results

### Resistance to apoptosis

**Figure 1:**
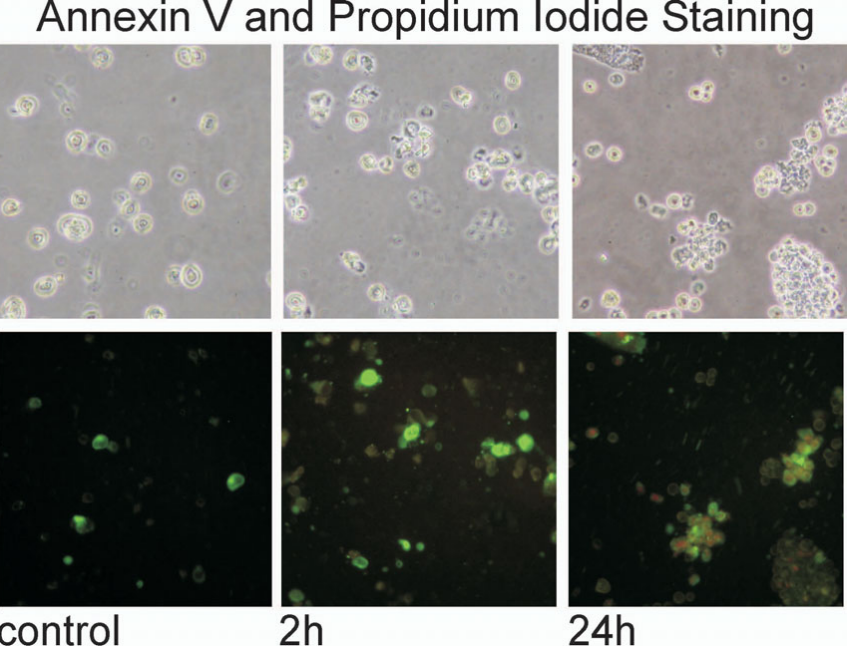
**Melanoma Cells are resistant to Curcumin induced Apoptosis**. Fluorescence micrographs of annexin V and propidium iodide stained human melanoma cells show that Curcumin induces only weakly apoptosis in M14 cells as evidenced by a very faint annexin V positive (green) staining. After 24 h of Curcumin treatment only very few M14 cells reach irreversible apoptosis and thereby a propidium iodide positive stage (red).

Human M14 melanoma cells treated for up to 24 h with 25 μM Curcumin show only very weak signs of apoptosis. Flow cytometry analysis reveal that only a small proportion of melanoma cells undergo early apoptosis in terms of translocation of the membrane phospholipid phosphatidylserine from the inner to the outer leaflet of the plasma membrane, where it becomes accessible to annexin V staining (green). Additionally less than 1% of the cells undergo irreversible apoptosis visualized here by propidium iodide staining (red). In consequence the staining intensities for both annexin V and propidium iodide are very weak indicating that the melanoma cells are very resistant to Curcumin in terms of apoptosis (figure 1). Only very few cells reach late apoptosis as indicated by weak propidium iodide staining. In contrast to the here presented data on melanoma cells, the human breast cancer cells MDA-MB-231 cells, who respond very well to Curcumin, undergo early and late apoptosis (published by us previously [3].

### Curcumin has no Effect on NFκB Signaling in Melanoma Cells

**Figure 2:**
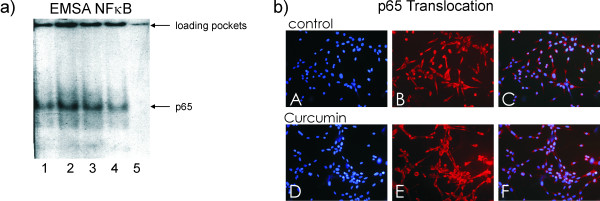
**Curcumin does not down-regulate NFκB in Melanoma cells**. a) Analysis of the binding of nuclear proteins from M14 melanoma cells to an oligo specific to the p65 binding site present in various promoters by EMSA revealed that Curcumin treatment did not alter p65 activity. There was no clear difference between the bands of treated (lane 2: 2 h; lane 3: 4 h; lane 4: 6 h) and untreated (lane 1) M14 cells using 10 μg of nuclear protein incubated with the ^32^P-labeled oligonucleotide. b) Immunofluorescent NFκB translocation assays using specific antibodies against the p65 unit show that NFκB is located both in the nucleus and the cytoplasm without any difference between Curcumin treated (lower panel) and untreated (upper panel) cells. (A, F: DAPI; B, G: p65; C, H: DAPI&p65). Experiments were performed in triplicates. Magnification ×200

NFκB signaling pathway is an important signal transduction pathway that plays a critical role in cell survival and apoptosis. As we observed only weak effects of Curcumin on apoptosis in M14 cells we investigated the status of NFκB/p65 in these cells after treatment with Curcumin. Nuclear extracts from Curcumin treated and untreated M14 cells were applied to electrophoretic mobility shift assays (EMSA) where the binding of transcription factors is revealed by the retarded electrophoretic migration of radioactively labeled oligonucleotides of the specific binding sequence (figure 2a). The specificity of the binding was assessed by addition of a 50 fold excess of cold oligonucleotides that abolished the band shifts observed (figure 2a; lane 5). Addition of an unrelated mutant oligonucleotide had no effect on NFκB binding (data not shown).

Curcumin treatment for 2, 4, and 6 h of M14 cells (figure 2a, lanes 2, 3, and 4,) does not show any reduction in the specific bands for the transcription factor NFκB in comparison to untreated cells (lane 1) indicating that Curcumin has no effect on the NFκB pathway in these cells.

Localization of the NFκB subunit p65 was monitored by immunofluorescence analyses using specific antibodies (figure 2b). M14 cells did not respond to Curcumin treatment and p65 was located in the nucleus as well as in the cytoplasm without any difference between treated and untreated control cells. DNA in the nuclei was stained with DAPI (figure 2b pictures A and D)

### CXCL1 is highly expressed in many primary and metastatic melanomas

**Figure 3:**
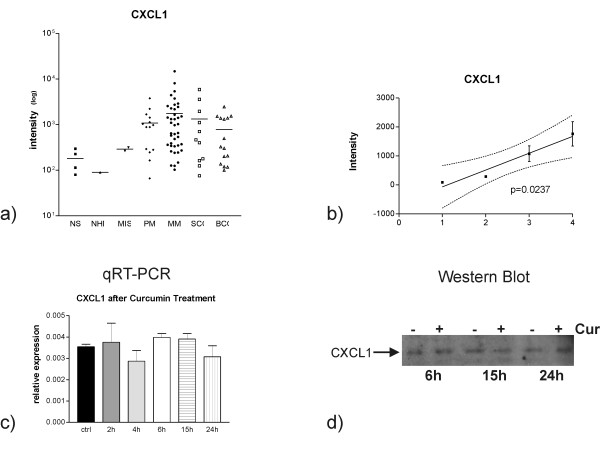
**a) Gene expression values of CXCL1 in normal skin (NS), normal human melanocytes (NHM), melanomas in situ (MIS), primary (PM) and metastatic (MM) melanomas were obtained from oligonucleotide microarray data**. The expression values of single samples (symbols) and the mean expression level (lines) are reported. b) CXCL1 expression during progression from normal human melanocytes (1) to melanoma *in situ *(2), primary (3) and metastatic (4) melanoma. There is a significant trend to increased CXCL1 expression during melanoma progression. c) qRT-PCR analysis of M14 melanoma cells shows that treatment with Curcumin for several time periods (2, 4, 6, 15 and 24 h) did not effect CXCL1 expression. d) Well in line with RT-PCR results, CXCL1 expression visualized here by Western Blots of cell culture supernatants was not altered in M14 cells treated for several time intervals (6 h, 15 h, 24 h) with Curcumin (lanes indicated with "+") versus respective un-treated (lanes indicated with "-") cells. Equal amounts of total protein were applied per lane and experiments were performed in triplicate.

We previously published, that the metastasis-related pro-inflammatory cytokines CXCL1 and -2 are down-regulated upon Curcumin treatment. The direct correlation between NFκB and CXCL1 and -2 expressions implies that the two cytokines are NFκB targets [4]. CXCL1 is also present in several metastasis signatures [26,36,37]. We wished to understand whether CXCL1 may play a role in melanoma so we analyzed published microarray gene expression data [35]. CXCL1 is strongly and highly differentially expressed in primary and metastatic melanoma and in other skin cancers (figures 3a and 3b). During the progression from normal human melanocytes (n = 1) over melanoma *in situ *(n = 2) and primary melanoma (n = 14) to metastatic melanoma (n = 41) there is a significant trend of increase of CXCL1 gene expression (p = 0.0237).

Given the potential role in melanoma progression, we analyzed whether the lack of effect of Curcumin on NFκB is transferred to the downstream target, CXCL1.

As determined by qRT-PCR, treatment of M14 melanoma cells with Curcumin for several time periods (2, 4, 6, 15 and 24 h) did not affect CXCL1 expression (figure 3c). CXCL2 was not expressed in M14 melanoma cells (data not shown). These results demonstrate that M14 cells are resistant to Curcumin on the level of transcriptional regulation by NFκB as well as on the level of its downstream targets.

These results were true also for the respective protein level. Western Blot results (figure 3d) revealed that M14 melanoma cells treated for several time periods (6 h, 15 h, 24 h) with Curcumin (lanes indicated with "+") did not express and secrete less CXCL1 into their conditioned media than their respective un-treated control cells (lanes indicated with "-").

Thereby we were able to demonstrate that Curcumin does not alter the expression of CXCL1 neither on transcript nor on protein level.

### Expression of multidrug resistance genes and cellular transporters

**Figure 4:**
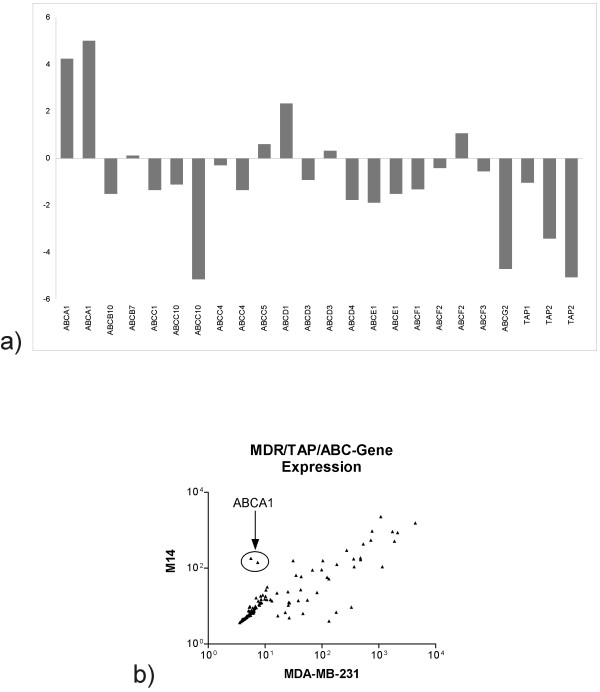
**Expression of ABC genes in M14 cells as compared to the Curcumin sensitive cell line MDA-MB-231**. Gene expression was analyzed using Affymetrix HU133plus2 arrays in M14 and MDA-MB-231 cells. The ratio of intensity levels of all probesets encoding ABC genes above the arbitrary threshold of 100 in M14 versus MDA-MB-231 cells were plotted on a log2 scale (A). All probesets encoding for ABC genes were used for a correlation plot (B).

In order to identify Curcumin resistance mechanisms we compared expression levels of known multi drug resistance genes and cellular transporters under the hypothesis, that Curcumin is rapidly removed from the resistant cells. For this purpose we performed microarray gene expression profiling using Affymetrix whole genome arrays (HGU133plus2) for M14 cells as well as for the strongly Curcumin sensitive breast cancer cell line MDA-MB-231. We identified differential expression for several ATP-binding cassette transporter genes that are expressed above the intensity threshold of 100 (figure 4a, data are shown for all probesets for each gene) and for correlated expression levels in the two cell lines (figure 4b). Over-expression of these genes may determine drug resistance and therefore, we tried to identify those genes that are over-expressed in M14 cells. ABCA1, a transporter that is involved in the cellular lipid removal pathway is the most highly over-expressed transporter in M14 cells as compared to Curcumin sensitive MDA-MB-231 cells. Over-expression is revealed by both probesets specific for this gene. ABCA1 is among the genes that are differentially expressed in the two cell lines in a statistically significant manner as analyzed by a permutation (Signficance Analysis of Microarray; [34]).

### Involvement of ABCA1 in Curcumin induced resistance

After we had seen that ABCA1 is among the most differentially expressed genes between non-resistant MDA-MB-231 and resistant M14 cells, we analyzed the functional involvement of this ATP-binding cassette transporter in the Curcumin induced resistance on NFκB signaling and apoptosis.

**Figure 5:**
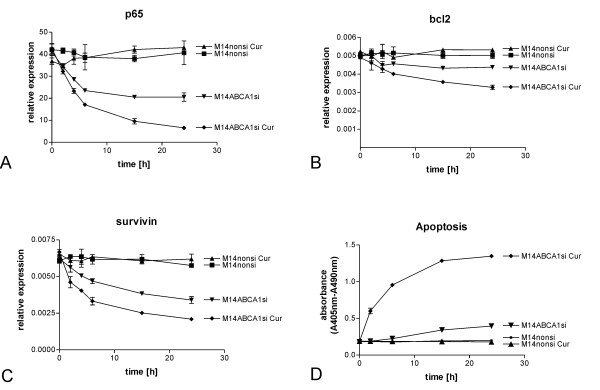
**Involvement of ABCA1 in Curcumin induced resistance**. The effect of Curcumin on p65 (A), the apoptosis related factors bcl2 (B) and surviving (C) and cell death (increase of free nucleosomes, D) was analyzed in a series of double modulation experiments. Silencing of the multidrug resistance gene ABCA1 led to a reduction of p65 expression down to 50% of the original level already after 15 h (panel A, M14ABCA1si (triangle down)). The simultaneous treatment with 25 μM Curcumin further reduced p65 expression down to about 15% of the initial value after 24 h treatment (panel A, M14ABCA1si Cur (diamond)). Expression of the NFκB regulated anti-apoptotic factors bcl2 and survivin was reduced after ABCA1 silencing: bcl2 expression was reduced 10% in ABCA1-silenced cells 24 h after transfection with siRNA (panel B, M14ABCA1si (triangle down)). Treatment of M14 cells carrying the ABCA1 knock-downs with 25 μM Curcumin led to a significant reduction of bcl2 expression of about 35% with (panel B, M14ABCA1si Cur (diamond)). Hence Curcumin reduces bcl2 expression in M14 cells transfected with ABCA1 siRNAs by about 25%. Expression of survivin was diminished upon silencing of ABCA1 in M14 cells to about 50% of the basic value 24 h after transfection with siRNAs (panel C, M14ABCA1si (triangle down)). The addition of 25 μM Curcumin to ABCA1 silenced cells further reduced survivin expression significantly in M14 cells down to about 40% of initial expression levels (figure 5C, M14ABCA1si Cur (diamond)). Accordingly Curcumin treatment diminishes survivin expression in ABCA1 silenced M14 cells to 10%. Apoptosis as measured by means of increased numbers of free nucleosomes in cell lysates was induced in ABCA1 silenced M14 cells 24 h after transfection with siRNAs (panel D, M14ABCA1si (triangle down)). Curcumin treatment of the ABCA1 silenced M14 cells dramatically induced apoptosis already after 2 h with increasing effect up to 24 h (panel D, M14ABCA1si Cur (diamond)). Transfection of an unspecific nonsilencing siRNA control alone (M14nonsi) or in combination with 25 μM Curcumin (M14nonsi Cur) did neither alter the expression of p65, bcl2 and survivin nor induce apoptosis in M14 cells (panels A, B, C, D, M14nonsi (triangle up), M14nonsi Cur (square))

We transiently knocked down ABCA1 in M14 cells by siRNA induced gene silencing and studied the effect of Curcumin on p65 (figure 5A), the apoptosis related factors bcl2 (figure 5B) and survivin (figure 5C) and cell death (increase of free nucleosomes, figure 5D) in a series of double modulation experiments.

Silencing of the multidrug resistance gene ABCA1 led to a reduction of p65 expression down to 50% of the original level already after 15 h (figure 5A, M14ABCA1si (tringle up)). The simultaneous treatment with 25 μM Curcumin further reduced p65 expression down to about 15% (p < 0.01) of the initial value after 24 h treatment (figure 5A, M14ABCA1si Cur (diamond)). It is clearly visible that ABCA1 silencing restores the effect of Curcumin on NFκB p65 subunit expression. Approximately 40% of this effect can be attributed to Curcumin. Yet ABCA1 silencing alone also reduces p65 expression, however, this effect is statistically not significant (p > 0.05).

Well in line with the effect of Curcumin on p65, expression of the NFκB regulated anti-apoptotic factors bcl2 and survivin was impaired after ABCA1 silencing. Bcl2 expression was reduced 10% in ABCA1-silenced cells 24 h after transfection with siRNA (figure 5B, M14ABCA1si (triangle down)). Treatment of M14 cells carrying the ABCA1 knock-downs with 25 μM Curcumin led to a significant inhibition of bcl2 expression of about 35% with p < 0.001 (figure 5B, M14ABCA1si Cur (diamond)). Hence Curcumin reduces bcl2 expression in M14 cells transfected with ABCA1 siRNAs by about 25%. Expression of survivin was diminished upon silencing of ABCA1 in M14 cells to about 50% of the basic value 24 h after transfection with siRNAs (figure 5C, M14ABCA1si (triangle down)). The addition of 25 μM Curcumin to ABCA1 silenced cells further reduced survivin expression significantly in M14 cells down to about 40% (p < 0.01) of initial expression levels (figure 5C, M14ABCA1si Cur (diamond)). Accordingly Curcumin treatment diminishes survivin expression in ABCA1 silenced M14 cells to 10%.

Apoptosis as measured by means of increased numbers of free nucleosomes in cell lysates was induced in ABCA1 silenced M14 cells 24 h after transfection with siRNAs (figure 5D, M14ABCA1si (triangle down)). Curcumin treatment of the ABCA1 silenced M14 cells dramatically induced apoptosis already after 2 h with increasing effect up to 24 h with a p-value of p < 0.01 (figure 5D, M14ABCA1si Cur (diamond)).

Transfection of an unspecific nonsilencing siRNA control alone (M14nonsi) alone or in combination with 25 μM Curcumin (M14nonsi Cur) did neither alter the expression of p65, bcl2 and survivin nor induce apoptosis in M14 cells (figure 5A, B, C, D, M14nonsi (triangle up), M14nonsi Cur (square))

Statistical evaluation of the results concerning the effects of ABCA1 on response to Curcumin (summarized in table 1) revealed that knocking-down the multidrug resistance gene alone already inhibits expression of the NFκB subunit p65 and the survival factors bcl2 and survivin and induces apoptosis in melanoma cells. However these effects are not statistically significant. Only the combination of knocking down ABCA1 together with simultaneous application of Curcumin results in a statistically highly significant response in terms of apoptosis.

**Table 1:** Statistical evaluation of the results concerning the effects of ABCA1 on response to Curcumin

**p65**				
Bonferroni's Multiple Comparison Test	Mean Diff.	t	P value	95% CI of diff
435nonsi vs 435nonsi Cur	1,393	0,2931	P > 0.05	-11.03 to 13.81
435nonsi vs 435ABCA1si	12,22	2,57	P > 0.05	-0.2006 to 24.64
435nonsi vs 435ABCA1si Cur	18,4	3,872	**P < 0.01**	5.985 to 30.82
				
**bcl2**				
Bonferroni's Multiple Comparison Test	Mean Diff.	t	P value	95% CI of diff
M14nonsi vs M14nonsi Cur	-0,00009025	0,4383	P > 0.05	-0.0006282 to 0.0004477
M14nonsi vs M14ABCA1si	0,0004488	2,18	P > 0.05	-0.00008914 to 0.0009868
M14nonsi vs M14ABCA1si Cur	0,0009655	4,689	**P < 0.001**	0.0004275 to 0.001503
				
**Survivin**				
Bonferroni's Multiple Comparison Test	Mean Diff.	t	P value	95% CI of diff
M14nonsi vs M14nonsi Cur	-0,00006799	0,1184	P > 0.05	-0.001568 to 0.001432
M14nonsi vs M14ABCA1si	0,001349	2,349	P > 0.05	-0.0001513 to 0.002849
M14nonsi vs M14ABCA1si Cur	0,002271	3,955	**P < 0.01**	0.0007706 to 0.003770
				
**Apoptose**				
Bonferroni's Multiple Comparison Test	Mean Diff.	t	P value	95% CI of diff
M14nonsi vs M14nonsi Cur	0,000869	0,005529	P > 0.05	-0.4193 to 0.4210
M14nonsi vs M14ABCA1si	-0,07965	0,5067	P > 0.05	-0.4998 to 0.3405
M14nonsi vs M14ABCA1si Cur	-0,688	4,377	**P < 0.01**	-1.108 to -0.2679

Knocking-down the multidrug resistance gene alone already inhibits expression of the NFκB subunit p65 and the survival factors bcl2 and survivin and induces apoptosis in melanoma cells. However these effects are not statistically significant. Only the combination of knocking down ABCA1 together with simultaneous application of Curcumin results in a statistically highly significant response in terms of apoptosis.

Taken together, resistance of M14 cells to Curcumin can be overcome by silencing of the ATP-binding cassette transporter ABCA1, which in turn leads to a reduction of p65, bcl2 and survivin and, as a consequence, to an induction of apoptosis. This is the first indication that transporters involved in the lipid metabolism are linked to NFκB and cell survival.

## Discussion

Among the plant derived products, Curcumin is perhaps the most interesting for long term anti-cancer chemoprevention. Much evidence has accumulated on the preventive effects of the polyphenol and the molecular pathways of its actions have been unraveled to considerable detail. Anti-proliferative and pro-apoptotic effects are most likely linked to the inhibition of pro-inflammatory and anti-apoptotic NFκB signaling, a now well established function of the compound. We have recently shown that Curcumin inhibits the formation but not the growth of metastases in an *in vivo *model of immunodeficient mice [3]. However, the anti-metastatic activity of the natural polyphenol cannot be completely ascribed to its pro-apoptotic activity. Curcumin apparently also influences a network of pro-metastatic players that depend on NFκB and in this context the inflammatory cytokines CXCL1 and -2 (GROα and β) appear to play a crucial role inasmuch as they influence the expression of several other pro-metastatic genes including CXCR4 [4]. The importance of CXCL1 in the process of metastasis has recently been reported and is also reflected by its presence in several metastasis signatures [26,37]. The lung metastasis signature presented by Minn and coworkers has been developed using a CXCL1 and -2 positive cell line, yet in human breast cancers, CXCL1 and -2 are not expressed at considerable levels, and in only a small minority of cases [37].

We show here that CXCL1 is strongly (and highly variably) expressed in human primary and metastatic melanomas as well as other skin cancers and several other groups have underlined the importance of CXCL1 in melanoma progression pointing to its tumorigenic and angiogenic effects [38] and to tumor-host interactions [39]. With this in mind we set out to test the potential of Curcumin as chemopreventive drug for melanoma.

Our results demonstrate that the highly metastatic melanoma cell line M14 is, however, relatively resistant to the effects of Curcumin, in particular if compared to what we have observed for breast cancer cells [3,4]. Even after 48 hours only early apoptosis can be observed and very few cells are irreversibly committed to cell death. This clearly correlates with the lack of effects on NFκB/p65 translocation and DNA binding capacity to the NFκB promoter presence in nuclear extracts. As a consequence, we saw, that expression of CXCL1 is not affected by the polyphenol. To our best knowledge, this is the first description of resistance to Curcumin, a drug that has itself been reported to be able to overcome drug resistance to classical chemotherapy [40-43]. Drug resistance is in most cases a result of over-expression of cellular transporters that are able to extrude the compound in a short time thereby avoiding the accumulation within the cell to active concentrations [28]. We therefore created and analyzed gene expression profiles of M14 cells in comparison to the highly sensitive cell line MDA-MB-231. Most of the gene expression differences between the two cell lines can be attributed to their different origin of tissue; yet we considered multiple drug resistance genes that are over-expressed in M14 cells as compared to the breast cancer cell line to be the reason for the resistance against Curcumin. Thereby we were able to identify essentially a single ATP-binding cassette transporter gene, ABCA1 to be responsible for the lack of response to Curcumin in M14 melanoma cells. Moreover we have previously analyzed the breast cancer cell lines MCF-7 and MDA-MB-468 and found that both, similar to MDA-MB-231 cells, are Curcumin sensitive and do not express ABCA1 (our unpublished data).

Here we show that silencing of ABCA1 by small interfering RNAs restores Curcumin sensitivity in M14 cells in terms of apoptosis, proliferation, transcriptional effects on NFκB/p65 transcription and CXCL1 expression in M14 cells.

ABCA1 is a lipid transporter that plays an important role in cholesterol efflux and thereby prevents toxicity associated with cholesterol overload. Moreover its activity is essential for formation of high-density lipoprotein (HDL) particles *in vivo *and mutations of ABCA1 lead to Tangier disease, an autosomal recessive trait characterized by HDL deficiency [44,45]. Low plasma HDL is associated with type 2 diabetes and metabolic syndrome [46,47] suggesting that ABCA1 contributes to this complication. Metabolic syndrome and type 2 diabetes are strongly correlated with therapy resistance and increased cancer risk [48]. We show here that the ability of Curcumin to induce apoptosis and to act on NFκB is restored by knocking down ABCA1 in treatment resistant M14 melanoma cells. Interestingly, silencing of the ABCA1 transporter alone, in the absence of Curcumin already induces apoptosis, yet to a lesser, statistically not significant extent. Future studies will show whether over-expression of the transporter and eventually others of the family in therapy sensitive tumor cells, activate the NFκB pathway and whether this might contribute to a more aggressive growth of multiple drug resistant cancers.

We show here that drug resistance is an important issue also for chemoprevention and future clinical trials with Curcumin should consider testing of ABCA1 expression as a potential response predictor. This is particularly important since the introduction of Curcumin into clinical prevention strategies most probably starts from the accumulation of data on effects in late stage disease patients who might carry metastases that over-express ABCA1. In the majority of primary and metastatic melanomas ABCA1 is highly expressed [35]. Melanoma is therefore most likely not a good target for Curcumin treatments unless patients are selected on the base of ABCA1 expression.

## Abbreviations

ABCA1: ATP-binding cassette, subfamily A, member 1; CXCL1: Chemokine (C-X-C motif) ligand 1; CXCL2: Chemokine (C-X-C motif) ligand 2; DAPI: 4',6-Diamidino-2-phenylindol; EMSA: electrophoretic mobility shift assay; HDL: high density lipoprotein; HPRT: Hypoxanthin-Phosphoribosyl-Transferase; NFκB: nuclear factor κB; RPII: RNA polymerase II.

## Competing interests

The authors declare that they have no competing interests.

## Authors' contributions

BB participated in designing the study, designed all experiments, participated in carrying out the molecular studies, analyzed and interpreted the data, supervised other co-authors performing molecular studies, participated in performing the statistical analysis, wrote the manuscript

CI, PK, EK, and GA, participated in carrying out the molecular studies, analyzed and interpreted the data.

VM carried out the bioinformatical analysis of the arrays and participated in interpreting the data

MJ and AN participated in designing the study and writing the manuscript

UP participated in designing the study, analyzed and interpreted the data, supervised other co-authors performing molecular studies, participated in performing the statistical analysis, participated in writing the manuscript

All authors read and approved the final manuscript

## Supplementary Material

Additional file 1 **Supplementary Table_Primer List_Bachmeier**. this file contains primer sequences Click here for file
