# Supplementary material for: Overexpression of the ATP binding cassette gene ABCA1 determines resistance to Curcumin in M14 melanoma cells
Source: Mol Cancer. 2009 Dec 23;8:129. doi: 10.1186/1476-4598-8-129 (PMC2804606; doi:10.1186/1476-4598-8-129)
Supplement: Additional file 1 — Supplementary Table_Primer List_Bachmeier. this file contains primer sequences [file 1476-4598-8-129-S1.DOC]

**Supplementary Table: Primer Sequences**

| **CXCL1** | |  |
| --- | --- | --- |
| sense | 5’-CTTGCCTCAATCCTGCATC-3’ | |
| antisense | 5’-CCTTCTGGTCAGTTGGATTTG-3’ | |
| **p65** | | |
| sense | 5’-ACGAGCTTGTAGGAAAGGACTG-3’ | |
| antisense | 5’-ATAGGAACTTGGAAGGGGTTGT-3’ | |
| **bcl-2** | | |
| sense | 5’-TGTGGATGACTGAGTACCTG-3’ | |
| antisense | 5’-AGAGACAGCCAGGAGAAATC-3’ | |
| **Survivin** | | |
| sense | 5’-ACTGAGAACGAGCCAGACTT-3’ | |
| antisense | 5’-CGGACGAATGCTTTTTATGTTC-3’ | |
| **RPII** | | |
| sense | 5’-GCACCACGTCCAATGACAT-3’ | |
| antisense | 5’-GTGCGGCTGCTTCCATAA-3’ | |
| **HPRT** | | |
| sense | 5’-CTCAACTTTAACTGGAAAGAATGTC-3’ | |
| antisense | 5’-TCCTTTTCACCAGCAAGCT-3’ | |
